# General Anaesthesia and Emergency Surgery in Heart Transplant Recipient

**DOI:** 10.1155/2015/256465

**Published:** 2015-12-16

**Authors:** Farshid Ejtehadi, Sharon Carter, Lucy Evans, Mubashar Zia, Howard Bradpiece

**Affiliations:** ^1^Department of General Surgery, The Princess Alexandra Hospital, Essex CM20 1QX, UK; ^2^Anesthetic Department, The Princess Alexandra Hospital, Essex CM20 1QX, UK

## Abstract

The number of patients who undergo heart transplant is increasing. Due to surgical emergencies, many of those may require general anesthesia in hospitals where subspecialized anesthetists may not be available. We present a case of a male patient who had heart transplant and required general anesthesia for emergency appendicectomy. Physiology of the heart after transplant, preoperative considerations, and postoperative monitoring has been discussed in our report.

## 1. Introduction

With an increasing number of patients in the general population with a heart transplant, many of these patients are presenting with acute surgical pathologies which may require surgical intervention [1]. These patients can pose a new challenge due to immunosuppression and altered cardiac physiology; therefore they require further preoperative assessment and optimization as well as intraoperative monitoring.

Previous research has shown that the clinical presentation of acute appendicitis is similar to those of the normal population. However, there is evidence that there is an increased rate of complications and hospital stay.

## 2. Case Summary

A 58-year-old man presented to accident and emergency complaining of one-day history of lower abdominal pain localised to the right iliac fossa, raised temperature, and vomiting. His past medical history included heart transplant 14 years ago following two myocardial infarctions at the ages of 43 and 44 along with a diagnosis of ischaemic cardiomyopathy. A recent echocardiogram performed four months prior to this admission showed normal left ventricular size and function, mild right ventricular impairment, and no documented valvular abnormalities. He is a current smoker of less than 10 cigarettes a day; treatment for hypercholesterolemia and medications included lisinopril, simvastatin, and oral immunosuppressive medications (azathioprine and tacrolimus). There was no significant family history and he was not known to have any allergies. On examination his baseline observations (blood pressure, heart rate, oxygen saturations, and temperature) were all within normal limits and he was haemodynamically stable. Abdominal examination revealed a mildly distended abdomen with lower abdominal tenderness on the right side. There was no clinical sign of peritonism.

Blood results including full blood count, renal profile, inflammatory markers, and clotting were all within normal range. On admission, ECG was in sinus rhythm at 78 bpm. Computerised tomography showed acute appendicitis; however there were no features suggestive of intra-abdominal collection or perforation. The patient was started on intravenous antimicrobial treatment prior to surgery.

A preoperative anaesthetic assessment was performed; airway assessment was mallampati 3 with good neck mobility and mouth opening. He reports the ability to walk on flat ground as well as up a flight of stairs without exhibiting chest pain or shortness of breath; he was classified as American Society of Anesthesiologists (ASA) 3.

Prior to surgery, the case was discussed with the patient's local transplant team, particularly with regard to his immunosuppression medication. It was advised that azathioprine be maintained at the same dose but converted to intravenous administration and that tacrolimus be administered sublingually. Additional administration of antifungal agents or commencing steroids was not advised.

Standard monitoring (according to AAGBI guidance) was established as well as invasive blood pressure monitoring, urinary catheter, central venous pressure, and oesophageal Doppler to monitor vital organ function and guide intravenous fluid therapy.

Anaesthesia was induced using midazolam 5 mg, fentanyl 100 mcg, and propofol 90 mg, followed by atracurium 50 mg once loss of consciousness occurred. He was intubated easily and a size-eight endotracheal tube was inserted and secured. During intubation, systolic blood pressure (SBP) was between 92 and 108 mmHg and 200 mcg of metaraminol was administrated.

Anaesthesia was maintained using sevoflurane, oxygen, and air mixture. He was ventilated using pressure controlled ventilation mode.

Intraoperatively he was given morphine 10 mg, ondansetron 4 mg, and dexamethasone 6.6 mg and required additional doses of atracurium to maintain muscle relaxation. Total of 1400 mcg of metaraminol in 6 divided doses was administrated.

The patient was haemodynamically stable throughout the procedure. Systolic blood pressure (SBP) was in the range of 80–115 mmHg, heart rate in the range of HR 62–85 bpm, and CVP in the range of 10–19 throughout the operation. Prior to extubation, BP of 90/45 mmHg with HR of 70 bpm and postextubation BP of 133/70 mmHg and HR of 79 bpm were recorded. Neuromuscular block was not reversed at the end of the procedure.

Initially, laparoscopic approach to abdominal cavity was attempted but due to intra-abdominal adhesion and inability to clearly identify detailed anatomy of the appendix and caecum the operation was converted to an open procedure to facilitate excision of the appendix. Appendix was safely divided near the base and removed through McBurney incision intraoperatively. He remained haemodynamically stable throughout the procedure and regular blood gases taken were unremarkable. At the end of the procedure he was extubated uneventfully and admitted to the postanaesthesia care unit (PACU) for initial postoperative care, later taken to high dependency unit (HDU) for further care and monitoring.

The patient developed a mild postoperative ileus; however, otherwise he made an unremarkable recovery. His length of stay was six days and there were no major complications within the 30-postoperative-day period. Histological examination confirmed appendicitis.

## 3. Discussion

It is important to have a good understanding of the change in physiology of patients with a heart transplant. There are key factors which must be addressed including the pharmacological management of the denervated heart, change in haemodynamic status, and prevention of transplant rejection postoperatively [1].

The transplanted heart does not have sensory sympathetic and parasympathetic innervation; it therefore has a higher resting heart rate of 90–110 bpm secondary to the loss of vagal tone. The resting ECG is commonly altered showing two p waves: one is from the recipients' own sinoatrial node and the other is the donors' sinoatrial node [2]. Patients are at higher risk of developing atrial flutter or atrial fibrillation. The patients' own SA node, although still functional, has no effect on the transplanted heart because the impulses cannot be conducted through the surgical suture lines. In the case of hypovolemia, a normal heart will increase its cardiac output by stimulating neurohormonal pathways resulting in increased heart rate and contractility; the transplanted heart cannot do this and is said to be “preload dependent” [3] as cardiac output becomes dependent on venous return [4, 5]. It has been shown that the transplanted heart may reinnervate over time [6, 7].

It is crucial to ensure that the patient is fully optimized preoperatively. An echocardiogram should be performed prior to surgery, ensuring assessment of ventricular and valvular function, establishing the patients exercise tolerance by assessing stress test findings and if necessary obtaining a review by a cardiologist [8]. It is important to assess patients for signs of organ rejection which include shortness of breath, fever, anuria, fatigue, fluid retention resulting in weight gain, and cardiac allograft vasculopathy [9]. Our patient only exhibited a fever but showed no temperature spikes during this admission. The risk of rejection is greatest in the first year after heart transplantation; our patient's transplantation was 13 years ago.

It was ensured that immunosuppressant medications (azathioprine and tacrolimus) were continued throughout his admission to reduce the risk of organ rejection.

Considering this gentleman's acute presentation not all the baseline investigations were obtained prior to surgery. Invasive monitoring was established to ensure tight control of his blood pressure, getting accurate readings with an arterial line and hence avoiding hypotension, vasodilation, and acute decrease in preload. Strict aseptic technique was maintained during the insertion of both the arterial and central lines to prevent infection and risk of transplant rejection. Such monitoring is also important to monitor haemodynamic changes during laryngoscopy and tracheal intubation. In heart transplanted patients laryngoscopy and tracheal intubation may not produce a sympathetic response secondary to the loss of cardiac baroreceptor reflexes.

In the transplanted heart, there is no alteration to heart rate in response to certain drugs including muscle relaxants (pancuronium and gallamine), anticholinergics (atropine and glycopyrrolate), and anticholinesterases (neostigmine and pyridostigmine). It is therefore important to consider other drugs that can be used in emergency situations, such as having ephedrine and isoprenaline [2, 4, 9].

Posttransplant patients are started on immunosuppressive therapy to prevent organ rejection. The most common drug regimen includes tacrolimus, mycophenolate, and prednisone. It is important to understand the action of these drugs and the impact they may have on the delivery of anaesthesia [4].

Tacrolimus acts by inhibiting T-lymphocyte activation as well as inhibiting IL-2 gene expression in T helper cells. It has multiple side effects which can have consequences for anaesthesia: hypertension, diabetes, neurotoxicity, and renal insufficiency [3, 10]. Prednisone has a similar side effect profile; however its action is different to tacrolimus because it has anti-inflammatory effect on organ systems. Mycophenolate mofetil is an inhibitor of inosine monophosphate dehydrogenase as well as having cytostatic effects on T- and B-lymphocytes. It also has implications for anaesthesia as it results in anaemia, leukopenia, and thrombocytopenia [3, 9, 10].

The patient was taking his immunosuppressants for many years prior to this presentation, which increases his risk for developing an infection. It has been advised that such patients should be started on steroids or that their steroid dose be increased. In this case neither were done upon advise from his transplant centre.

## 4. Summary

- To have an understanding that the transplanted heart has no sensory sympathetic and parasympathetic innervation makes them prone to developing atrial fibrillation and atrial flutter.
- The transplanted heart is “preload dependent”; therefore it is important to maintain a sufficient systolic pressure and prevent hypovolemia.
- The transplanted heart does not respond to muscle relaxants, anticholinergics, and anticholinesterases; therefore in emergency situations ephedrine and isoproterenol can be used.
- To be aware of the side effect profile of immunosuppressive therapy and how this may affect the anaesthetic agents given is necessary.
- It is crucial to continue immunosuppressive therapy, considering other routes if necessary.
